# Optimization of Preimplantation Genome Profiling Supports Genomic Selection in Cattle

**DOI:** 10.3390/cells15080705

**Published:** 2026-04-16

**Authors:** Shihui Yan, Saina Yan, Yuanweilu Cheng, Hengyuan Cui, Yang Pang, Jingfang Si, Li Jiang, Dongxiao Sun, Alfredo Pauciullo, Johannes A. Lenstra, Shenming Zeng, Yi Zhang

**Affiliations:** 1 State Key Laboratory of Animal Biotech Breeding, National Engineering Laboratory of Animal Breeding, Key Laboratory of Animal Genetics and Breeding of Ministry of Agriculture and Rural Affairs, College of Animal Science and Technology, China Agricultural University, Beijing 100107, China; bs20253040572@cau.edu.cn (S.Y.); cywl@cau.edu.cn (Y.C.); cuihy0910@163.com (H.C.); mie18530682607@163.com (Y.P.); sijingfang@cau.edu.cn (J.S.); lijiang@cau.edu.cn (L.J.); sundx@cau.edu.cn (D.S.); 2 College of Biological Sciences, China Agricultural University, Beijing 100107, China; yansaina99@cau.edu.cn; 3 Department of Agricultural, Forest and Food Science, University of Torin, 10124 Torino, Italy; alfredo.pauciullo@unito.it; 4 Faculty of Veterinary Science, Utrecht University, 3584 CL Utrecht, The Netherlands; j.a.lenstra@uu.nl

**Keywords:** whole-genome amplification, genotyping, embryo, genomic selection, bovine

## Abstract

Preimplantation embryo genomic selection (eGS) enables selection prior to implantation and could accelerate genetic gain in cattle. A major hurdle is the limited DNA from embryo biopsies, requiring efficient whole-genome amplification (WGA) for accurate genomic analyses. However, alternative WGA methods and genotyping strategies have not been systematically compared in cattle. This study evaluated different methods for WGA (multiple displacement amplification (MDA) or multiple annealing and looping-based amplification cycles (MALBAC)) and for genotyping (single nucleotide polymorphism array (SNP-array), genotyping by targeted sequencing (GBTS), or whole-genome sequencing (WGS)) using 3-, 6-, and 9-cell bovine samples. MDA consistently outperformed MALBAC across various performance metrics, including amplification length, call rates, genome coverage (93.43–94.40% vs. 53.01–67.08%), and genotyping concordance (0.89–0.98 vs. 0.75–0.92). GBTS achieved the highest call rates, while SNP-array and GBTS showed excellent concordance and low error rates. WGS provided genome-wide data for precise aneuploidy detection. We further validated the workflow in trophectoderm biopsies and arrested embryos, generating reliable data for genomic evaluation, sex determination, and aneuploidy screening. MDA from ≥6 cells combined with GBTS or SNP-array showed a favorable balance of efficiency and accuracy for bovine eGS. This framework may facilitate the application of eGS in cattle breeding by enhancing selection intensity and accelerating genetic improvement.

## 1. Introduction

Over the past decade, genomic selection (GS) has revolutionized animal breeding by enabling the prediction of breeding values based on genome-wide markers [1]. To further enhance selection intensity and reduce costs, preimplantation embryo genomic selection (eGS) has emerged as a promising strategy. By integrating reproductive technologies, such as embryo transfer and in vitro fertilization, with the genomic tools, eGS facilitates the genotyping of early-stage embryos and the estimation of embryo genomic estimated breeding values (eGEBVs) prior to implantation [2]. Unlike conventional postnatal GS, eGS allows for the simultaneous evaluation of multiple half- and full-sibling embryos, substantially increasing selection intensity and accelerating genetic gain [3,4]. However, the limited DNA content in embryo biopsies (~6 pg per cell) poses a critical technical bottleneck, necessitating robust whole-genome amplification (WGA) techniques tailored for low-input samples [5].

WGA non-selectively amplifies DNA from minute quantities of material, enabling downstream genomic analyses [6]. In livestock, the most widely adopted WGA methods are multiple displacement amplification (MDA) and multiple annealing and looping-based amplification cycles (MALBAC). MDA utilizes phi29 DNA polymerase for high-fidelity, isothermal strand displacement amplification [7], while MALBAC employs random primers in a quasi-linear pre-amplification step, followed by limited-cycle PCR to reduce amplification bias and improve genome coverage [8]. Although both methods are extensively applied in human preimplantation genetic testing (PGT), their relative performance has remained largely unexplored [9,10,11]. MDA has been employed in bovine embryos WGA, with amplified DNA used for genomic analyses relevant to eGS [12,13,14]. Nevertheless, MALBAC remains underutilized in cattle and has not been compared directly with bovine cells.

Genotyping platforms for WGA products must balance accuracy, cost, and throughput. The single nucleotide polymorphism array (SNP-array) is widely used in bovine GS due to its low cost and standardized protocols, enabling the reliable detection of common variations in embryo biopsies [15,16,17,18]. However, their reliance on predefined markers limits the detection of structural variants. Whole-genome sequencing (WGS) offers high-resolution detection of SNPs, insertions and deletions (indels), and copy number variations (CNVs), but its application in bovine eGS is constrained by cost and incomplete coverage [19,20,21,22]. Genotyping by target sequencing (GBTS) bridges this gap by combining probe-based capture with high-depth sequencing, enabling flexible and cost-effective genotyping of the target regions [23]. It has been used for genome-wide association studies and GS in cattle [24,25], but not yet for bovine eGS.

In this study, the bovine mammary epithelial (MAC-T) cell line was employed as a biologically relevant and controllable model to systematically evaluate the impact of low numbers (3, 6, and 9) of cells, and to compare the performance of two WGA methods (MDA and MALBAC) and three genotyping platforms (SNP-array, GBTS, WGS) on genomic analysis accuracy. On the basis of amplification efficiency, genotype call rate, genome coverage, genotyping concordance, error rate, and aneuploidy detection, we optimized the workflow for low-input bovine cells, which was validated for actual bovine embryos and may very well be essential for the use of eGS in cattle breeding programs.

## 2. Materials and Methods

### 2.1. Experimental Design

Using the MAC-T cell line as the experimental model, we prepared triplicates of low-input samples (3, 6, or 9 cells) as well as a triplicate of bulk samples containing approximately 70,000 cells. Low-input samples underwent WGA via MDA and MALBAC, while bulk samples provided reference genomic DNA (gDNA). Amplified DNA and reference gDNA were analyzed using SNP-array, GBTS, and WGS. Key performance metrics included genotype call rate, genome coverage, genotyping concordance, error rate, amplification bias, and number of SNPs. The optimized workflow was applied to actual bovine embryos for genomic evaluation, sex determination, and preimplantation genetic testing for aneuploidy (PGT-A) (Figure S1).

### 2.2. Cell Culture and Isolation

The MAC-T cell line was provided by the National Engineering Laboratory of Animal Breeding [26]. MAC-T cells were cultured in DMEM supplemented with 10% fetal bovine serum and antibiotics, maintained at 37 °C in a humidified incubator containing 5% CO_2_. Cells were passaged regularly when confluence levels exceeded 85%. After three passages, individual cell groups consisting of 3, 6, or 9 cells were manually isolated using a micropipette, with three replicates per group. The remaining cells were used for gDNA extraction. All samples were stored at −20 °C until use.

### 2.3. Embryo Sample Collection and Processing

Holstein embryos used in this study were produced in vitro, and oocytes were collected from slaughterhouse-derived ovaries. Under a stereomicroscope, cumulus–oocyte complexes (COCs) with homogeneous cytoplasm and at least three compact layers of cumulus cells were selected for in vitro maturation. Selected COCs were cultured in a maturation medium at 38.5 °C under 5% CO_2_ for 22–24 h. After maturation, in vitro fertilization was performed. Sperm were prepared by Percoll density-gradient centrifugation and adjusted to a final concentration of 1 × 10^6^ sperm/mL, and then co-incubated with the oocytes for 16–18 h. After fertilization, the remaining cumulus cells were removed by gentle pipetting until complete denudation, and the presumptive zygotes were transferred into an IVC medium containing NaCl, KCl, CaCl_2_, NaHCO_3_, pyruvate, BSA, and amino acids for continued culture at 38.5 °C under 5% CO_2_, 5% O_2_, and 90% N_2_ until the blastocyst stage.

Trophectoderm biopsy was performed on in vitro-produced Holstein blastocysts. A total of 23 Day 7–8 blastocysts were selected. Using a laser-assisted zona drilling system (ILS-400M, Huayuexing Medical Technology Co., Ltd., Guangzhou, China), approximately 6–8 trophectoderm (TE) cells were isolated from each blastocyst. The biopsied cells were washed in PBS and transferred into 0.2 mL polypropylene tubes containing 4 μL of PBS (Invitrogen, Carlsbad, CA, USA) for subsequent whole-genome amplification.

In addition, 10 embryos that had arrested at the 8-cell stage during in vitro culture were collected. At the time of collection, the zona pellucida was removed using an acidic Tyrode’s solution, and each embryo was washed three times in PBS (Invitrogen) to remove the residual culture medium and any loosely attached cellular debris. The washed embryos were individually transferred into 0.2 mL polypropylene tubes containing 4 μL of PBS. All embryo samples were stored at −20 °C until further analysis.

### 2.4. Genomic DNA Extraction and WGA

Genomic DNA from all MAC-T low-input samples and embryos (23 TE biopsies and 10 arrested embryos) was amplified using the REPLI-g Single Cell Kit (#150343, Qiagen, Mississauga, ON, Canada), which is based on the MDA method. In parallel, low-input MAC-T samples were also WGA-amplified using the Single Cell Whole Genome Amplification Kit (#KT110700150, Yikon, Taizhou, Jiangsu, China), which employs the MALBAC method. Amplified DNA was purified with the QIAquick PCR Purification Kit (#28106, Qiagen, Chatsworth, CA, USA). After amplification according to the manufacturers’ protocols, DNA concentration and purity were quantified using a NanoDrop spectrophotometer (Thermo Fisher Scientific, Waltham, MA, USA), while integrity was assessed through a 1% agarose gel electrophoresis. gDNA from bulk samples was extracted using the Genomic DNA Extraction Kit (#DP304, TIANGEN, Beijing, China).

### 2.5. Genome-Wide Genotyping

Genome-wide genotyping was conducted using three platforms: a bovine SNP-array, GBTS, and WGS. The performance of these platforms was first evaluated using MAC-T cell line samples. Based on the initial results (detailed in the Section 3), GBTS was selected as the optimal method for the subsequent genotyping of all embryo samples.

For the SNP-array analysis, qualified DNA samples were genotyped using the Illumina GGP Bovine 100K Chip microarray (Illumina, San Diego, CA, USA) [27]. GBTS was based on targeted capture sequencing for genotyping [28]. Pre-hybridization libraries were prepared with the GenoBaits DNA Library Prep Kit (Molbreeding, Shijiazhuang, Hebei, China). Based on Qubit quantification, DNA concentrations ranged from 741.33 to 795.00 ng/μL for MDA products and from 48.40 to 57.37 ng/μL for MALBAC products. For target-capture library preparation, 50 μL of MDA products and 65 μL of MALBAC products were used as input. DNA was fragmented, end-repaired, and A-tailed, followed by adapter ligation, and PCR amplification under the following conditions: 98 °C for 45 s; 12 cycles of 98 °C for 15 s, 60 °C for 30 s, and 72 °C for 30 s; followed by 72 °C for 1 min and a 4 °C hold. PCR products were purified using GenoPrep DNA Clean Beads (Molbreeding). Capture enrichment employed GenoBaits Panel probes (Molbreeding) and was followed by sequencing on the DNBSEQ T7 platform (MGI Tech, Shenzhen, Guangdong, China) with paired-end 150 bp reads. Sequencing data underwent quality control via fastp (v0.20.1). Reads were aligned to the Bos taurus UMD 3.1 reference genome using Sentieon (v202010.02) with the BWA-MEM algorithm (default settings). Aligned reads were sorted using Sentieon (v202010.02). Duplicate sequences were removed using a Sentieon (v202010.02) driver, and the base quality score recalibration (BQSR) scores were calculated. gVCF files were generated using the Haplotyper module of Sentieon (v202010.02) driver, and multi-sample VCF datasets were produced with the GVCFtyper module. Genotypes for 139,097 core SNPs were extracted for downstream analyses. Detailed per-sample sequencing depth distribution and target-coverage metrics for these core SNPs are provided in Table S1.

For the WGS analysis, PE150 libraries were sequenced on the DNBSEQ T7 platform (MGI Tech), achieving average sequencing depths of 10× (low-input) and 20× (bulk). Raw data were processed with Trimmomatic (v0.39) to remove low-quality reads and adapters. The subsequent analytical steps mirrored the GBTS approach. Variants were filtered using BCFtools (v1.15.1) with the stringent parameters: “QD  <  2.0 || QUAL  <  30.0 || SOR  >  3.0 ||MQ  <  20.0 || FS  >  60.0 || MQ Rank Sum  <  −12.5 || ReadPos Rank Sum  <  −8.0”.

### 2.6. Genotyping Concordance Evaluation

Genotype data from MAC-T samples were analyzed using PLINK (v2.0) and custom R scripts (v4.3.0) to calculate relative call rates, genotyping concordance, and error rates, including allele dropout (ADO), heterozygosity gain, and homozygosity reversal [29]. The relative call rate is the proportion of loci detected in amplified samples relative to loci detected in gDNA. Genotyping concordance indicates the proportion of amplified loci that match those in gDNA. ADO represents the proportion of loci where the genotype is AB in gDNA appearing as AA or BB in amplified products. Heterozygosity gain refers to the proportion of loci where the genotype is AA or BB in gDNA appearing as AB in amplified products. Homozygosity reversal refers to the proportion of loci where the genotype is AA (or BB) in gDNA appearing as BB (or AA) after amplification.

For SNP-array and GBTS, the original call rate represented the proportion of loci detected relative to the total number. For WGS data, the GC content analysis utilized FastQC (v0.11.9), while sequencing depth, genome coverage, and mapping rate were assessed using SAMtools (v1.17).

### 2.7. Amplification Bias Analysis

To compare the amplification bias of the MDA and MALBAC methods, the average read depth was calculated for each consecutive, non-overlapping 1 Mb bin across the whole genome using SAMtools (v1.17). Estimated copy numbers (CNs) were calculated as twice the ratio of the average and median read depth [30]. Coefficients of variation (CVs) across the genome were calculated per window (1 kb, 10 kb, 100 kb, 1 Mb, 10 Mb, and 100 Mb) as the standard deviation of the read depth divided by the average value [31].

### 2.8. MAC-T Cell Line Aneuploidy Detection

Based on the WGS data of MAC-T cell gDNA, CNs across chromosomes 1–29 and X were assessed in 1 Mb bins. A CN > 2.6 was considered indicative of trisomy, while a CN < 1.4 was considered monosomy [32].

### 2.9. Genetic Analysis of Bovine Embryos

#### 2.9.1. Genomic Evaluation

Genomic evaluation of embryos was based on the genomic best linear unbiased prediction (GBLUP) method using a reference population of 19,000 Chinese Holstein cattle [33]. First, a genomic relationship matrix (G) was constructed using the SNP set from both the reference population and the embryos. SNPs with a call rate < 0.9 and a minor allele frequency (MAF) < 0.01 were excluded. Missing genotypes were then imputed to a standard 50K density (54,609 SNPs) using Beagle (v5.4) [34]. The imputed dataset underwent a second round of QC (MAF > 0.01 and Hardy-Weinberg equilibrium (HWE) test *p* > 1 × 10^−5^). Second, de-regressed proofs (DRP) for milk yield, fat percentage, and protein percentage were calculated using an iterative regression equation [35]. Third, direct genomic breeding values (DGV) for each trait were estimated using a univariate animal model in DMU (v6.5.2) [36]:

y=1μ+Zg+e

where y is the vector of DRP for milk production traits (milk yield, fat percentage, and protein percentage); μ is the overall mean; and 1 is a vector of 1 s. g is the vector of genomic breeding values, following a distribution $N\left(0,G\sigma_{g}^{2}\right)$, where $\sigma_{g}^{2}$ is the additive genetic variance, and G is the genomic relationship matrix (G matrix). The G matrix was calculated as $\frac{zz^{\prime }}{2\Sigma p_{i}\left(1-p_{i}\right)}$ [37], in which $p_{i}$ represents the MAF of marker i, and Z represents the MAF adjusted marker matrix with entries for genotypes AA and aa being $\left(0-{2p}_{i}\right)$ and $\left(2-{2p}_{i}\right)$, respectively. Z is a design matrix linking g to y, and e is the vector of random residual effects.

#### 2.9.2. Sex Determination

Embryo sex was determined by analyzing the heterozygosity of an X-chromosome SNP marker panel. SNPs were filtered by excluding those with a call rate < 0.9, a MAF < 0.015, or a heterozygosity > 0.01 in male embryos to remove pseudoautosomal region (PAR) SNPs [38]. Embryos were classified as male if their heterozygosity rate across the panel was ≤0.02 and as female if the rate exceeded this threshold [38].

#### 2.9.3. Aneuploidy Detection

Aneuploidy was assessed from embryo data via CNV analysis using CNVkit (v0.9.8) [39], which infers the genome-wide copy number alterations by calculating the sequencing depth ratio between target and reference regions [40]. An analysis was conducted using default parameters. Embryos were classified as trisomy for a chromosome if the inferred CN was >2.6, and monosomy if the CN was <1.4 [32]. All copy number profiles were visualized using R (v4.3.0).

### 2.10. Statistical Analysis

A one-way ANOVA was used to assess differences between WGA technologies under the same cell number for amplified DNA concentration, mapping rate, genome coverage, and GC content. Additional ANOVA analyses evaluated genotype call rate, genotyping concordance, error rates, and number of SNPs across cell numbers and genotyping platforms within each WGA strategy. Tukey’s HSD test facilitated multiple comparisons. Results were expressed as mean ± standard deviation, with statistical significance defined at *p* < 0.05.

## 3. Results

### 3.1. Amplification Efficiency

**Figure 1:**
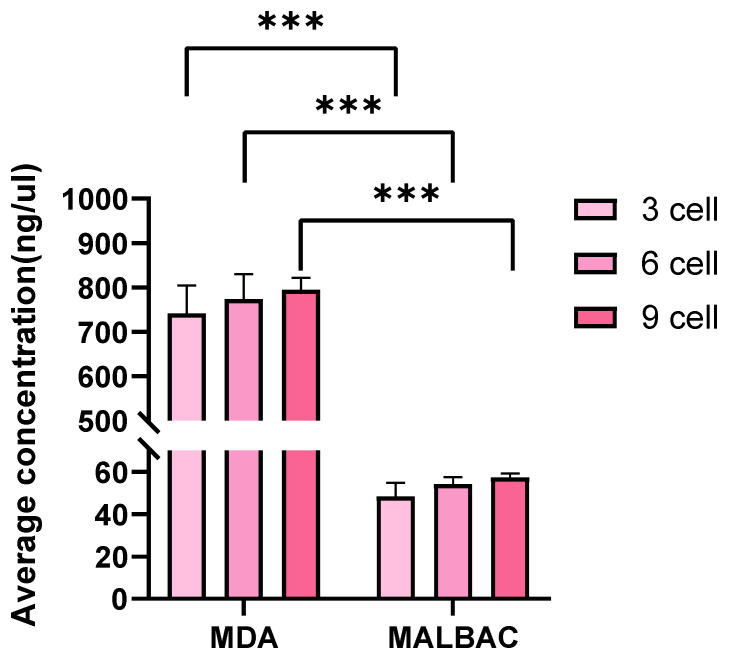
Comparative analysis of WGA efficiency for different cell inputs. Asterisks indicate significant differences between MDA and MALBAC within the same cell-number group (*** *p* < 0.001).

At the same cell inputs, DNA concentrations from MDA amplification were significantly higher than those from MALBAC (*p* < 0.05, Figure 1). Agarose gel electrophoresis showed that MDA amplification products exhibited a predominant band at ~15 kb, whereas MALBAC products ranged primarily between 0.25 and 2 kb (Figure S2). A higher DNA yield was observed with increasing cell numbers for both WGA methods, with the general pattern 9-cell > 6-cell > 3-cell (Figure 1).

### 3.2. SNP Calling Performance

#### 3.2.1. Two WGA Methods

**Figure 2:**
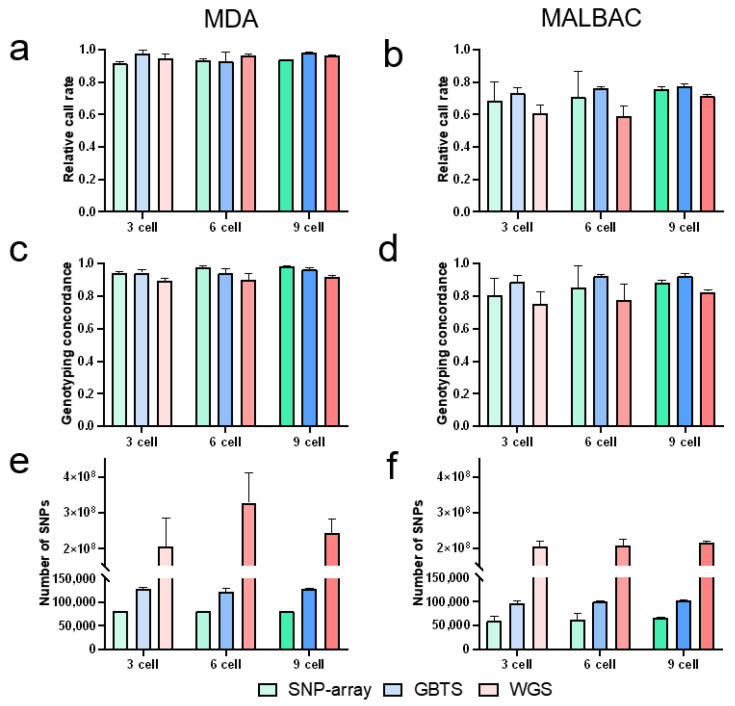
SNP calling performance across different cell inputs for MDA and MALBAC using SNP-array, GBTS, and WGS. Relative call rates for MDA (**a**) and MALBAC (**b**); genotyping concordance for MDA (**c**) and MALBAC (**d**); and number of SNPs detected for MDA (**e**) and MALBAC (**f**).

At equivalent cell inputs and genotyping techniques, MDA showed higher SNP relative call rates (0.92–0.98) than MALBAC (0.59–0.77) (Figure 2a,b). While MDA showed relatively stable call rates across cell numbers, MALBAC tended to show higher call rates at higher inputs, with the highest values observed in the 9-cell group (Figure 2a,b). Original call rates mirrored this trend, with MDA having higher original call rates than MALBAC (MDA: 88.80–97.00%; MALBAC: 66.13–76.37%) (Table 1). MDA maintained high original call rates (88.80–96.66%) at 3- and 6-cell inputs, whereas MALBAC showed a general increase from 66.13% (3-cell) to 76.37% (9-cell) (Table 1).

A genome coverage analysis confirmed that MDA had significantly higher genome coverage than MALBAC at matched cell numbers (93.43% vs. 54.40% for 3-cell, 94.40% vs. 53.91% for 6-cell, and 94.14% vs. 67.08% for 9-cell; *p* < 0.05, Table 2). Correspondingly, MDA-derived products yielded a higher number of SNPs, with minimal variability across cell numbers for both methods (Figure 2e,f). MDA also exhibited a GC content closer to the bulk reference (44.13%) than MALBAC (Table 2). However, mapping rates for both methods were in the same range (Table 2).

#### 3.2.2. Three Genotyping Platforms

Notable differences in SNP calling efficiency were observed among genotyping platforms. MDA, GBTS, and WGS outperformed the SNP-array in relative call rates (Figure 2a), while GBTS surpassed the SNP-array and WGS for MALBAC (Figure 2b). Increasing cell numbers improved the SNP-array and GBTS performance in MALBAC, but had minimal impact on MDA (Figure 2a,b). GBTS yielded significantly higher original call rates than the SNP-array for both WGA methods (*p* < 0.05, Table 1). Original call rates improved with cell inputs in MALBAC, rising from 66.1% (3 cells) to 72.8% (9 cells) for the SNP-array and from 72.5% (3 cells) to 76.4% (9 cells) for GBTS (Table 1).

### 3.3. Genotyping Concordance and Error Types

#### 3.3.1. Two WGA Methods

**Figure 3:**
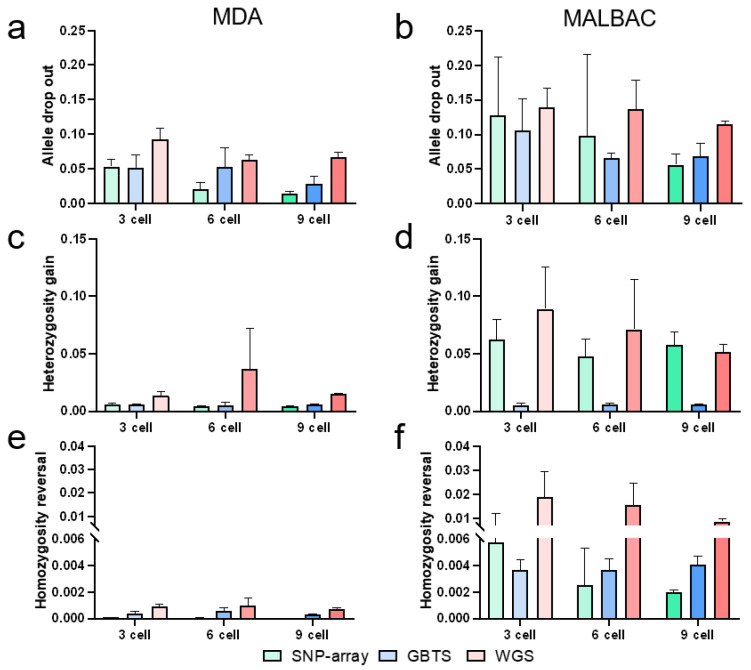
Genotyping error profiles across different cell inputs for MDA and MALBAC using SNP-array, GBTS, and WGS. Allele dropout frequency for MDA (**a**) and MALBAC (**b**); heterozygosity gain frequency for MDA (**c**) and MALBAC (**d**); and homozygosity reversal frequency for MDA (**e**) and MALBAC (**f**).

MDA showed consistently higher genotyping concordance (0.89–0.98) than MALBAC (0.75–0.92) across all conditions (Figure 2c,d). Concordance improved with cell numbers for both methods (Figure 2c,d). MDA exhibited lower frequencies of amplification errors, including ADO, heterozygosity gain, and homozygosity reversal, than MALBAC (Figure 3a–f). ADO was the dominant error type (48.11–91.33%) and decreased with cell inputs (Figure 3a,b). Heterozygosity gain (5.35–50.15%) and homozygosity reversal (0.02–7.23%) frequencies remained stable but improved marginally in MALBAC with higher cell numbers (Figure 3c–f).

#### 3.3.2. Three Genotyping Platforms

The SNP-array and GBTS showed a significantly higher concordance than WGS in MDA (*p* < 0.05, Figure 2c). For MALBAC, GBTS outperformed WGS (*p* < 0.05, Figure 2d), while the SNP-array showed a higher concordance than WGS (*p* = 0.14, Figure 2d). The concordance improved across all platforms with increasing cell numbers (Figure 2c,d). WGS exhibited the highest error frequencies (ADO, heterozygosity gain, and homozygosity reversal across all conditions) (Figure 3a–f), likely due to lower sequencing depth (mean 10×). The SNP-array showed a lower homozygosity reversal than GBTS in MDA (*p* < 0.05, Figure 3e), while GBTS exhibited a significantly lower heterozygosity gain than the SNP-array in MALBAC (*p* < 0.05, Figure 3d). ADO frequencies decreased with cell numbers across all platforms (Figure 3a,b). For MDA, heterozygosity gain and homozygosity reversal remained relatively stable (Figure 3c–e). In contrast, for MALBAC, WGS exhibited a gradual decrease, while GBTS remained stable (Figure 3d–f).

### 3.4. Amplification Bias

**Figure 4:**
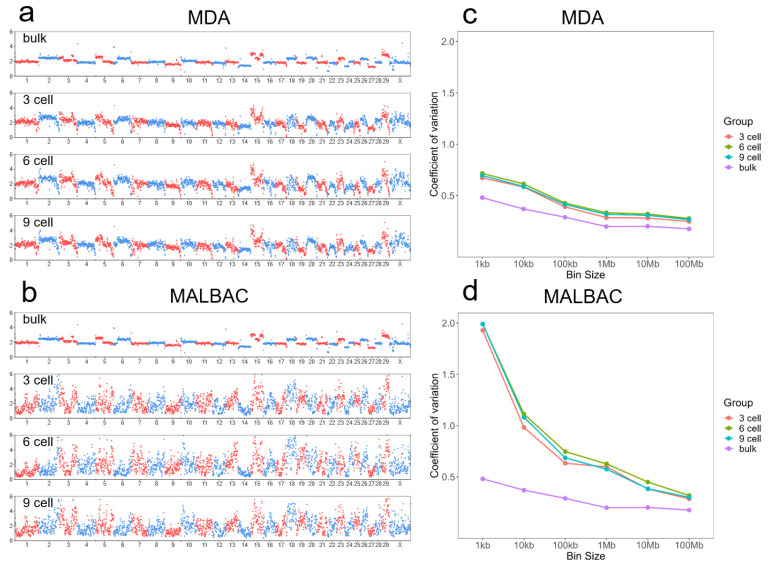
Amplification bias analysis for WGA methods. (**a**,**b**) Read depths across the genome with a 1 Mb bin size across different cell inputs for MDA and MALBAC. (**c**,**d**) Coefficient of variation for read depths along the genome as a function of bin sizes for MDA and MALBAC.

Amplification bias, evaluated through average read depths in 1 Mb genomic bins from WGS data of the MAC-T cell line, was consistently lower in MDA than MALBAC (Figure 4a,b). The CV of read depths decreased with larger bin sizes for both WGA methods, but remained significantly lower in MDA than in MALBAC (Figure 4c,d). At 1 Mb bin size resolution, CVs ranged between 0.29 and 0.33 for MDA versus 0.58 and 0.63 for MALBAC, indicating a reduced amplification bias for MDA (Figure 4c,d). Cell numbers (3, 6, and 9) had a negligible influence on the amplification bias (Figure 4c,d).

### 3.5. Aneuploidy Analysis in MAC-T Cell Line

**Figure 5:**
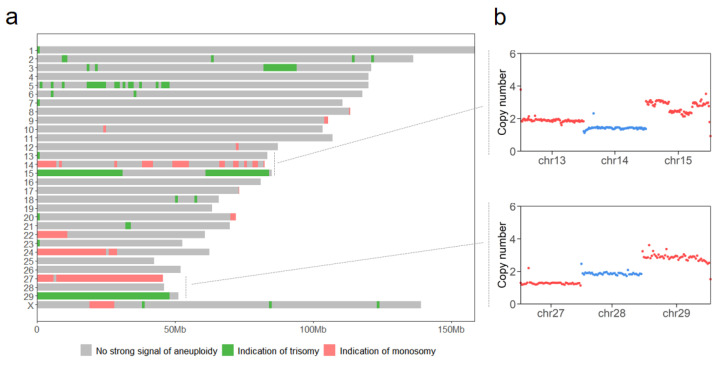
Aneuploidy analysis in the MAC-T cell line. (**a**) Genome-wide aneuploidy screening of the MAC-T cell line across chromosomes 1 to 29 and X. (**b**) Representative copy number variations (CNVs) in chromosomes 13, 14, 15, 27, 28, and 29.

A WGS-based aneuploidy analysis revealed extensive chromosomal abnormalities in the MAC-T cell line (Figure 5a). Monosomy was prevalent on chromosomes 14, 22, 24, and 27, with limited regions on chromosomes 9, 10, 12, 20, and X. Trisomy occurred extensively on chromosomes 15 and 29, alongside localized regions on chromosomes 2, 3, 5, 6, 18, 21, and X. Chromosomes 4, 11, 16, 19, 25, 26, and 28 exhibited no detectable aneuploidy. Representative CNV profiles for selected chromosomes are shown in Figure 5b.

### 3.6. Validation of the Optimized Workflow in Bovine Embryos

**Figure 6:**
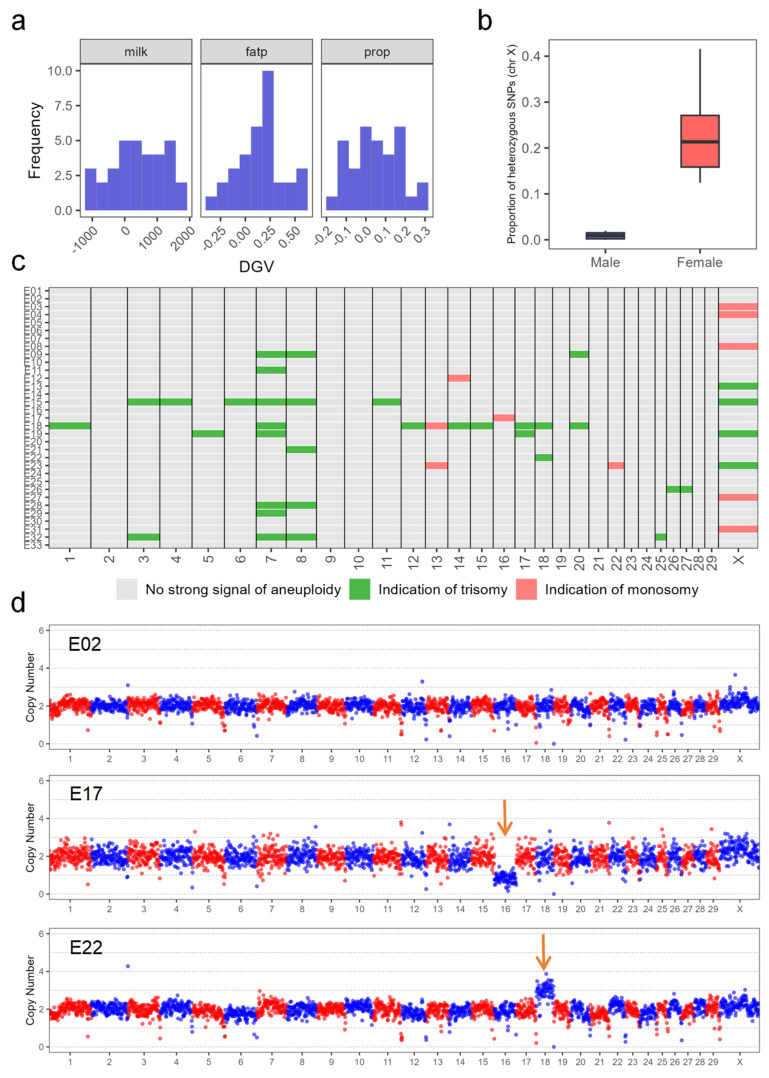
Genomic profiling of bovine embryos reveals variations in genomic values, sex, and aneuploidies. (**a**) Distribution of direct genomic values (DGV) for the indicated traits; (**b**) proportion of heterozygous single nucleotide polymorphisms (SNPs) on the X chromosome for male and female embryos; and (**c**) genome-wide aneuploidy per chromosome (1 to 29 and X) for embryo samples indicated at the left. Samples E18–E25 and E28–E29 are arrested embryos, whereas samples E01–E17, E26–E27, and E30–E33 are TE biopsy samples. (**d**) Representative copy number variation (CNV) profiles from PGT-A. E02: A euploid embryo with a normal diploid genome. E17: an embryo with a chromosome 16 monosomy. E22: An embryo with a chromosome 18 trisomy.

We validated the performance of the established low-input genomic detection workflow by conducting a comprehensive genetic analysis on 33 bovine embryos (Figure 6a–d). The original genotype call rates for these embryos ranged from 84.83% to 99.05%. Arrested embryos had lower original genotype call rates than TE biopsies, with mean values of 93.35% and 97.08%, respectively (84.83–98.47% for arrested embryos vs. 91.36–99.05% for TE biopsies). Application of the workflow enabled a successful genomic evaluation for three milk production traits. We observed a substantial difference in genetic value among the embryos, with DGV ranging from −1081.08 to 1717.39 for milk yield, −0.30 to 0.61 for fat percentage, and −0.17 to 0.29 for protein percentage (Figure 6a). For embryo sex determination, a panel of 4356 X-chromosome SNPs was used. Twenty male embryos exhibited a low X-chromosome heterozygosity of 0 to 0.02, clearly lower than the values for the remaining 13 female embryos (0.12 to 0.42) (Figure 6b).

An aneuploidy screening revealed that 20 out of the 33 embryos (60.61%) were aneuploid (Figure 6c). The aneuploidy rate was higher in the arrested embryos (70.00%, 7/10) than in the TE biopsies (56.52%, 13/23). Trisomies in 13 out of 20 aneuploid embryos were more frequent than monosomies (9 out of 20). Several embryos exhibited complex aneuploidies involving both trisomy and monosomy or multiple chromosomal abnormalities (Figure 6c,d).

## 4. Discussion

This study provides a systematic evaluation of WGA methods (MDA and MALBAC) and genotyping platforms (SNP-array, GBTS, and WGS) for low-input genomic analysis in cattle.

### 4.1. Amplification Performance of WGA Methods

MDA consistently outperformed MALBAC in DNA yield, fragment length, and amplification uniformity. This superior performance stems from MDA’s rolling-circle amplification mechanism mediated by phi29 DNA polymerase, which enables continuous synthesis of long DNA fragments (>10 kb) [7]. In contrast, MALBAC’s multiple primer annealing and cyclic amplification result in lower efficiency and higher bias [41], aligning with prior human cell line studies [30,31,42,43,44]. Notably, MDA’s ADO rate decreases at greater sequencing depths [31]. Consistent with this, we observed that in MDA-amplified 3-cell samples, the error rate decreased from 0.28 at 5× depth to 0.11 at 7×, 0.09 at 8×, and 0.08 at 9×. This indicates that the relatively low sequencing depth in our study was likely a contributing factor to the observed error rates, further suggesting that insufficient sequencing depth may lead to SNP genotyping errors.

An amplification bias analysis revealed higher biases in MALBAC across all cell number levels, coupled with elevated GC content deviations compared to MDA. These findings differ from prior studies conducted in droplets and tubes, where MALBAC demonstrated greater uniformity [44]. Furthermore, normalization strategies such as adjustments for sequencing depth and GC content may substantially mitigate amplification bias in MALBAC [30].

Higher initial cell numbers improved the amplification efficiency for both WGA methods. Notably, MDA produced a higher genotype call rate and greater concordance, as well as lower error rates, as cell numbers increased. It demonstrated a robust performance even with only 3 to 6 cells, a finding consistent with earlier studies [45,46,47].

### 4.2. Performance of Different Genotyping Platforms

Among the genotyping platforms, GBTS outperformed the SNP-array in original call rates (72.49–97.00% vs. 66.13–89.53%) due to its targeted enrichment and high-depth sequencing (>100×). In contrast, the SNP-array’s susceptibility to background noise reduced its original call rate [29].

A genotyping concordance analysis demonstrated distinct advantages for different platforms depending on the amplification approach used. For MDA, the SNP-array presented the highest genotyping concordance and lowest ADO frequency, whereas GBTS excelled in MALBAC. These findings are consistent with previous applications of SNP-arrays in WGA studies of human fibroblast cell lines [48]. The targeted enrichment of GBTS effectively mitigated the elevated error rates associated with MALBAC, thereby improving the overall genotyping accuracy [49]. Although WGS normally delivers the most comprehensive genotyping, it showed high error rates at a low depth (10×) and is currently less favorable for routine embryo screenings.

### 4.3. Analysis of Aneuploidy in the MAC-T Cell Line Based on WGS

WGS provided unique advantages, including a genome-wide amplification bias analysis and chromosomal aneuploidy detection [50]. Read depth variability (CV: 0.25–0.72 for MDA vs. 0.29–1.99 for MALBAC) confirmed amplification uniformity in MDA relative to MALBAC. In the MAC-T cell line, WGS revealed extensive aneuploidy, including trisomy on chromosomes 3, 5, 15, and 29, and monosomy primarily on chromosomes 14, 22, 24, and 27. These findings partially concur with earlier karyotypic analyses of MAC-T clonal cell lines [51]. In cattle, the incidence of chromosomally abnormal cells is higher after in vitro (72.00%) production than after in vivo production [52]. In laboratory cell lines, environmental stressors associated with in vitro culture conditions may further exacerbate chromosomal loss, duplication, and rearrangement, thereby contributing to the observed variability in aneuploidy across the cell line [53,54]. Such chromosomal instability may also complicate copy-number interpretations in MAC-T cells. In particular, subclonal heterogeneity within the cell population may cause the observed copy-number signals to represent averaged states across different karyotypic backgrounds, thereby attenuating the amplitude of some chromosomal gains or losses and increasing the complexity of copy-number profiles [51].

### 4.4. Application of the Low-Input Genomic Detection Workflow

The primary challenge in embryo genomic analysis stems from the extremely limited and heterogeneous nature of biopsy material [55,56]. This study was designed to optimize the workflow by using a MAC-T cell line to rigorously simulate low-input biopsy conditions (3, 6, and 9 cells) before applying it to embryos. This strategy identified a workflow combining MDA-based WGA with the GBTS platform as optimal, effectively balancing genome coverage, genotyping accuracy, and amplification bias under low-input conditions. The subsequent successful application of this optimized workflow to actual bovine embryos, including fragile TE biopsies and arrested embryos with compromised DNA, validates its robustness for diverse and challenging samples.

This integrated workflow addresses two key frontiers in cattle genetic improvement: eGS and PGT-A [18,57,58]. Unlike prior studies, which typically performed genetic analyses via SNP-array in separate experiments [14,59,60], our method consolidates genomic evaluation, sex determination, and PGT-A into a single and efficient pipeline.

For eGS, the workflow successfully generated reliable genotype data needed for the calculation of DGV, demonstrating that GBTS is an efficient and feasible alternative to conventional SNP-arrays [29,47]. For sex determination, embryo sex identification was achieved using a curated panel of X-chromosome SNPs. To our knowledge, this is the first successful implementation of embryo sex determination in cattle based on GBTS data, moving beyond the traditional reliance on PCR-based methods [61]. For PGT-A, the detected aneuploidy rate of 60.61% aligns with previous reports [60]. The results revealed biologically significant patterns: a higher aneuploidy rate in arrested embryos (70.00%) than in TE biopsies (56.52%), and a higher prevalence of trisomies over monosomies. These findings demonstrate that the sensitivity of the workflow is adequate for the detection of the genetic abnormalities that cause embryonic developmental failure [62,63], which is essential for reliable screening embryo with high developmental potential. A larger, more diverse embryo cohorts of embryo may further refine our protocol. For instance, WGS of embryos would allow the detection of smaller structural variations that affect the breeding values.

## 5. Conclusions

This study provides a comparative evaluation of WGA and genotyping strategies for low-input genomic analysis in cattle by assessing genome coverage, genotyping concordance, and amplification bias. Among the tested combinations, MDA from ≥6 cells combined with GBTS or SNP-array appeared to provide the most favorable balance between efficiency and accuracy for bovine eGS. Under the conditions tested, this workflow showed promising performance in bovine embryos for genomic evaluation, sex determination, and PGT-A. However, further validation will be necessary before routine implementation in cattle breeding programs.

## Figures and Tables

**Table 1:** Comparison of original call rates for MDA and MALBAC methods across different cell inputs assessed by SNP-array and GBTS.

WGA Method	Group	SNP-Array			GBTS		
		Number of Miss Loci	Number of Call Loci	Original Call Rate (%)	Number of Miss Loci	Number of Call Loci	Original Call Rate (%)
MDA	3 cell	10,123 ± 640	80,226 ± 640	88.80 ± 0.71 ^b^	4439 ± 2981	128,649 ± 2981	96.66 ± 2.24 ^a^
	6 cell	9866 ± 350	80,483 ± 350	89.08 ± 0.39 ^b^	10,912 ± 7960	122,176 ± 7960	91.80 ± 5.98 ^a^
	9 cell	9460 ± 74	80,889 ± 74	89.53 ± 0.08 ^b^	3990 ± 712	129,098 ± 712	97.00 ± 0.54 ^a^
MALBAC	3 cell	30,601 ± 10,064	59,748 ± 10,064	66.13 ± 11.14 ^b^	36,612 ± 4608	96,476 ± 4608	72.49 ± 3.46 ^a^
	6 cell	28,686 ± 13,484	61,663 ± 13,484	68.25 ± 14.93 ^b^	32,806 ± 1094	100,282 ± 1094	75.35 ± 0.82 ^a^
	9 cell	24,569 ± 1387	65,780 ± 1387	72.81 ± 1.54 ^b^	31,455 ± 2609	101,633 ± 2609	76.37 ± 1.96 ^a^

Values are presented as mean ± standard deviation. Within the same row, different superscript letters in the original call rate (%) columns indicate significant differences between the SNP-array and GBTS (*p* < 0.001). For each WGA method and genotyping platform, no significant differences were observed among cell-number groups (*p* > 0.05).

**Table 2:** Mean depth, mapping rate, genome coverage, and GC content across different cell inputs for MDA and MALBAC. The methods were evaluated by WGS.

WGA Method	Group	Mean Depth (X)	Number of Mapped Bases (bp)	Mapping Rate (%)	Genome Coverage (%)	GC Content (%)
MDA	3 cell	10.2 ± 4.0	203,963,408 ± 81,174,184	99.46 ± 0.48	93.43 ± 1.43 ^a^	41.31 ± 0.21 ^b^
	6 cell	15.3 ± 4.4	319,737,813 ± 93,161,567	97.00 ± 4.63	94.40 ± 0.25 ^a^	40.87 ± 0.07 ^b^
	9 cell	12.0 ± 2.0	242,537,557 ± 39,121,758	99.71 ± 0.19	94.14 ± 0.30 ^a^	41.53 ± 0.09 ^b^
MALBAC	3 cell	9.8 ± 1.0	204,066,870 ± 15,125,388	99.73 ± 0.20	54.40 ± 5.96 ^b^	47.15 ± 2.10 ^a^
	6 cell	9.5 ± 1.3	203,657,390 ± 19,026,158	98.39 ± 2.48	53.91 ± 8.15 ^b^	48.13 ± 1.83 ^a^
	9 cell	10.0 ± 0.3	214,939,525 ± 4,772,173	99.83 ± 0.06	67.08 ± 1.13 ^b^	47.27 ± 0.04 ^a^

Values are presented as mean ± standard deviation. For the same cell-number group, different superscript letters in the genome coverage (%) and GC content (%) columns indicate significant differences between MDA and MALBAC (*p* < 0.001). No significant differences were observed for mapping rate (*p* > 0.05).

## Data Availability

The original data presented in the study are openly available in CNSA (https://db.cngb.org/cnsa/ (accessed on 18 March 2026)) with accession number CNP0009176.

## Supplementary Materials

The following supporting information can be downloaded at: https://www.mdpi.com/article/10.3390/cells15080705/s1, Figure S1: Workflow of the experiment; Figure S2: Gel electrophoresis of amplification products for MDA and MALBAC for different cell inputs; Table S1. Per-sample sequencing depth distribution and target-coverage metrics for core target SNPs in GBTS.

## Abbreviations

The following abbreviations are used in this manuscript:

ADO	Allele dropout
CNs	Copy numbers
CNVs	Copy number variations
CVs	Coefficients of variation
DGV	Direct genomic breeding values
DRP	De-regressed proofs
eGEBVs	Embryo genomic estimated breeding values
eGS	Preimplantation embryo genomic selection
GBLUP	Genomic best linear unbiased prediction
GBTS	Genotyping by target sequencing
GS	Genomic selection
indels	Insertions and deletions
MAC-T	Mammary Alveolar Cells-large T antigen
MAF	Minor allele frequency
MALBAC	Multiple annealing and looping-based amplification cycles
MDA	Multiple displacement amplification
PGT	Preimplantation genetic testing
PGT-A	Preimplantation genetic testing for aneuploidy
SNP-array	Single nucleotide polymorphism array
TE	Trophectoderm
WGA	Whole-genome amplification
WGS	Whole-genome sequencing
