# Supplementary material for: Optimization of Preimplantation Genome Profiling Supports Genomic Selection in Cattle
Source: Cells. 2026 Apr 16;15(8):705. doi: 10.3390/cells15080705 (PMC13115129; doi:10.3390/cells15080705)
Supplement: Supplementary file 1 [file cells-15-00705-s001.zip › cells-4226934-supplementary.pdf]

Supplementary Materials:

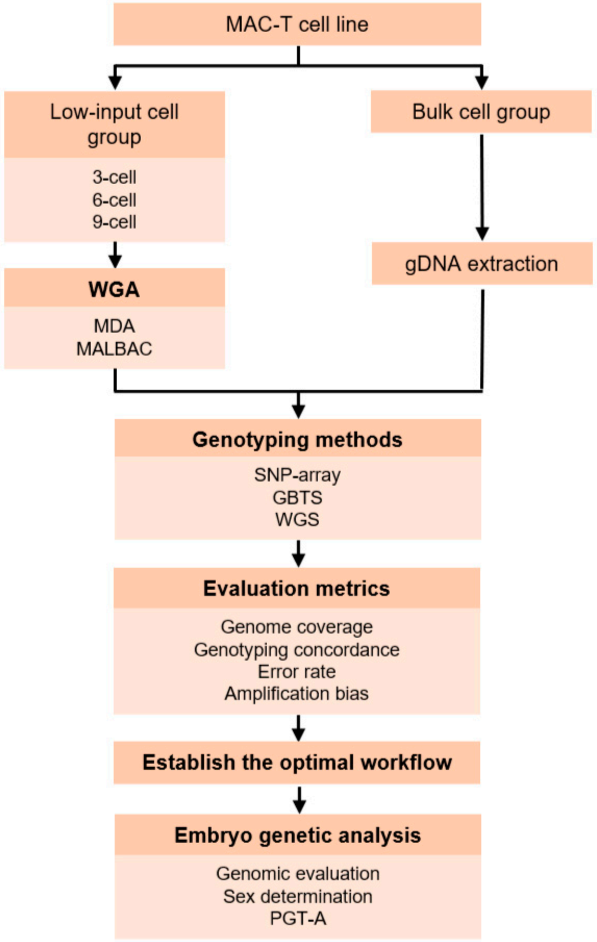

Figure S1. Workflow of the experiment.

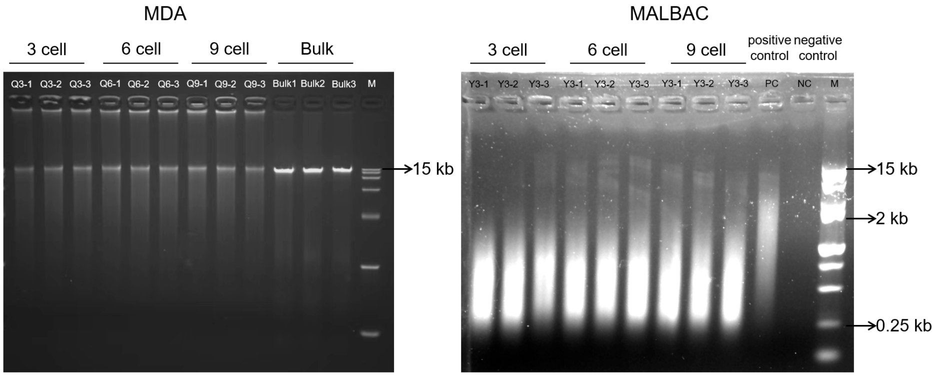

Figure S2. Gel electrophoresis of amplification products for MDA and MALBAC for different cell inputs.

Table S1. Per-sample sequencing depth distribution and target-coverage metrics for core target SNPs in GBTS.

WGA method	sample	Mean depth across core target SNPs (X)	Core target SNPs with depth ≥10% of mean depth (%)	Core target SNPs with depth ≥30% of mean depth (%)	Core target SNPs with depth ≥50% of mean depth (%)
MDA	3cell-1	167.9	97.73	85.52	68.66
	3cell-2	54.9	94.07	80.67	64.45
	3cell-3	100.2	95.10	80.30	63.64
	6cell-1	96.7	96.90	84.49	67.83
	6cell-2	47.3	92.67	78.78	64.17
	6cell-3	26.6	86.34	79.47	64.17
	9cell-1	72.7	97.40	87.38	70.07
	9cell-2	71.1	95.95	83.15	66.16
	9cell-3	47.3	97.26	88.06	72.65
MALBAC	3cell-1	138.1	63.60	43.83	34.00
	3cell-2	112.9	60.36	42.21	32.72
	3cell-3	108.6	60.76	43.94	34.95
	6cell-1	92.8	66.04	46.68	35.88
	6cell-2	133.7	63.09	43.23	33.73
	6cell-3	122.2	62.58	43.57	33.60
	9cell-1	94.5	65.78	46.38	36.06
	9cell-2	97.5	66.89	46.66	36.27
	9cell-3	128.6	66.37	45.01	34.52

Mean depth was calculated across all core target SNPs, including loci with zero coverage. Percentages indicate the proportions of core target SNPs with sequencing depths reaching at least 10%, 30%, or 50% of the sample-specific mean depth.
